# Effects of Topical Bimatoprost 0.01% and Timolol 0.5% on Circadian IOP, Blood Pressure and Perfusion Pressure in Patients with Glaucoma or Ocular Hypertension: A Randomized, Double Masked, Placebo-Controlled Clinical Trial

**DOI:** 10.1371/journal.pone.0140601

**Published:** 2015-10-20

**Authors:** Francesco Oddone, Luca Rossetti, Lucia Tanga, Francesca Berardo, Manuela Ferrazza, Manuele Michelessi, Gloria Roberti, Gianluca Manni, Marco Centofanti

**Affiliations:** 1 Clinical and Research Institute of Ophthalmology IRCCS Fondazione G. B. Bietti, Via Livenza 3, 00198, Rome, Italy; 2 Clinica Oculistica, Dipartimento di Scienze della Salute, University of Milan, San Paolo Hospital, Via Antonio di Rudinì, 8 20142, Milan, Italy; 3 Clinical Science and Translational Medicine Department, University of Rome Tor Vergata, Viale Oxford 81, 00133, Rome, Italy; Eye Institute of Xiamen University, CHINA

## Abstract

**Purpose:**

To compare the 24-hour (24h) effects on intraocular pressure (IOP) and cardiovascular parameters of timolol 0.5% and bimatoprost 0.01% in open angle glaucoma and ocular hypertensive subjects.

**Methods:**

In this prospective, randomized, double masked, crossover, clinical trial, after washout from previous medications enrolled subjects underwent 24h IOP, blood pressure (BP) and heart rate (HR) measurements and were randomized to either topical bimatoprost 0.01% at night plus placebo in the morning or to timolol 0.5% bid. After 8 weeks of treatment a second 24h assessment of IOP, BP and HR was performed and then subjects switched to the opposite treatment for additional 8 weeks when a third 24h assessment was performed. The primary endpoint was the comparison of the mean 24h IOP after each treatment. Secondary endpoints included the comparisons of IOP at each timepoint of the 24h curve and the comparison of BP, HR, ocular perfusion pressure and tolerability.

**Results:**

Mean untreated 24h IOP was 20.3 mmHg (95%CI 19.0 to 21.6). Mean 24h IOP was significantly lower after 8 weeks of treatment with bimatoprost 0.01% than after 8 weeks of treatment with timolol 0.5% bid (15.7 vs 16.8 mmHg, p = 0.0003). Mean IOP during the day hours was significantly reduced from baseline by both drugs while mean IOP during the night hours was reduced by -2.3 mmHg (p = 0.0002) by bimatoprost 0.01% plus placebo and by -1.1 mmHg by timolol 0.5% bid (p = 0.06). Timolol 0.5% significantly reduced the mean 24h systolic BP from baseline, the diastolic BP during the day hours, the HR during the night hours, and the mean 24h systolic ocular perfusion pressure.

**Conclusion:**

Both Bimatoprost 0.01% and Timolol 0.5% are effective in reducing the mean 24h IOP from an untreated baseline but Bimatoprost 0.01% is more effective than timolol 0.5% throughout the 24h. Timolol 0.5% effect on IOP is reduced during the night hours and is associated with reduced BP, HR and ocular perfusion pressure.

**Trial Registration:**

EU Clinical Trial Register and EudraCT# 2010-024272-26

## Introduction

Lowering intraocular pressure (IOP) is the only evidence based method available to treat glaucoma and it has been shown in large randomized clinical trials that each mmHg of IOP reduction is associated with a reduction of the risk of visual field damage progression [1–4].

Medical therapy is widely accepted as the first step in the management of glaucoma and among the pharmacological agents available prostaglandin and prostamide analogues and beta-blockers are widely used as first line drugs for their IOP lowering efficacy and overall safety profile.

Nevertheless differences do exist between these agents in terms of IOP lowering effect and safety profiles and cost-effectiveness [5], and the knowledge of these differences is important for individualizing the treatment strategy for the single glaucoma patient.

Among topical beta-blockers Timolol maleate is the most widely used and its efficacy and safety have been thoroughly studied since its first introduction in the medical therapy of glaucoma in the late 70s. Timolol 0.5% is able to reduce the IOP on average by 20–25% [6] with 2 daily administrations 12 hours apart. Its topical tolerability profile is very good while issues arise when considering the systemic safety. In fact timolol is contra-indicated in individuals suffering by sinus bradycardia, heart block or cardiac failure and chronic obstructive pulmonary disease. Moreover the use of beta-blockers in patients with glaucoma has been reported to be associated with the reduction of blood pressure (BP) and heart rate (HR) [7,8].

Bimatoprost, a prostamide analogue, has been shown to be effective in lowering the IOP by 30–35% at the concentration of 0.03% [9,10] and to be associated with local adverse effects including conjunctival hyperemia, eyelash growth and skin pigmentation. Recently, a new formulation of bimatoprost with a reduced concentration of the active drug (0.01%) has been made available for treating glaucoma, and it showed a similar ocular hypotensive efficacy compared to the 0.03% formulation and an improved local tolerability profile [11].

It has been shown by several studies that the IOP in healthy individuals undergo physiological diurnal and nocturnal variations with peaks and troughs that can vary according to the population being studied or to the methodology of IOP measurement being used. These variations have been found more pronounced in untreated patients with glaucoma and are subject to modifications induced by the topical hypotensive therapy [12]. Similarly also the blood pressure varies physiologically in the 24h and its variations may be influenced by systemic and topical therapies.

Despite several reports have been published about the efficacy and safety of topical timolol and bimatoprost in comparison with other drugs, only one single-masked parallel study compared the 24h effect of timolol 0.5% and bimatoprost 0.03% on IOP and no 24h direct comparisons are available involving bimatoprost 0.01%. [13] Moreover little information is available coming from direct comparisons regarding the effects of these pharmacological agents on cardiovascular parameters in the 24h.

The purpose of the present study is to directly compare the 24 hour ocular hypotensive efficacy and cardiovascular effects of bimatoprost 0.01% with those of timolol 0.5% in open angle glaucoma and ocular hypertensive subjects naïve to treatment or washed-out from previous topical medications.

## Methods

This prospective, randomized, double-masked, cross-over clinical trial (registered with EudraCT # 2010-024272-26, https://eudract.ema.europa.eu, and available in the EU Clinical Trials Register, https://www.clinicaltrialsregister.eu) was carried out between February 2011 and January 2012 at the Glaucoma Unit of the IRCCS Fondazione G.B.Bietti, Rome (Italy) and the authors confirm that all ongoing and related trials for this drugs are registered. Newly diagnosed or previously treated glaucoma or ocular hypertensive patients after adequate washout were enrolled. The study protocol was approved by the IRCCS Fondazione G.B.Bietti Ethical Committee named "ASL Roma A” and was in accordance with the principles of the declaration of Helsinki and all patients signed a written informed consent before enrollment.

Inclusion criteria were a diagnosis of primary open angle glaucoma or ocular hypertension based on the 4th edition of the European Glaucoma Society Guidelines criteria, an IOP <27 mmHg for untreated newly diagnosed patients or IOP<22 mmHg for patients on treatment with any hypotensive medication in monotherapy and <27 mmHg after adequate washout (see below). The cut-off point of 27 mmHg was introduced as a safety criterium for glaucoma patients in order to maximise the likelyhood of having IOPs on treatment below 21 mmHg assuming a minimum hypotensive efficacy of the study drugs of 25% from untreated baseline IOP values. Washout IOP had to be >21 mmHg for ocular hypertension subjects while no lower limit of untreated IOP was set for glaucoma patients.

Exclusion criteria were defined as age < 18 years, any past or active ocular disease other than glaucoma, closed/barely open anterior chamber angle or history of acute angle closure, any cause of secondary elevation of IOP, argon or selective laser trabeculoplasty within the last six months, any previous ocular surgery, ocular inflammation/infection occurred within three months prior to the pre-trial visit, uncontrolled systemic diseases that might require initiating or altering concomitant use of systemic medications that can interfere with the study drugs or with IOP (e.g. beta-adrenergic antagonists, alpha-adrenergic agonists, calcium channel blockers, ACE inhibitors and angiotensin II receptor antagonists), hypersensitivity to benzalkonium chloride or to any other component of the trial drug solutions, contraindications to the study drugs including reactive airway disease, chronic obstructive pulmonary disease, sinus bradycardia, second or third degree atrioventricular block, overt cardiac failure, or cardiogenic shock, severe allergic rhinitis and bronchial hyperreactivity, pregnancy, nursing or childbearing potential without using adequate contraception.

### Study plan and randomization

Treatment naive patients and previously treated patients after adequate washout from previous medications and who fulfilled the eligibility requirements detailed above were included in this study.

Washout time varied according to the ongoing treatment and was 6 weeks for prostaglandin/prostamide analogues, 4 weeks for beta-blockers and alpha-agonists, 2 weeks for carbonic anhydrase inhibitors and miotics [6].

At baseline enrolled patients were admitted and performed a 24h IOP, BP and HR curve and were then randomized, according to a 1:1 computer-generated randomization code list, to be treated with either bimatoprost 0.01% administered once at night (08.00pm) + placebo administered once in the morning (at 8.00am) or timolol 0.5% administered twice daily (8.00am and 8.00pm). After 8 weeks of the first treatment a second 24h IOP, BP, and HR curve was performed and then patients were crossed-over to the opposite treatment regimen (patients on bimatoprost 0.01% + placebo switched to timolol 0.5% twice daily and vice versa) and were treated for 8 additional weeks. At the end of the follow-up (16 weeks from baseline and 8 weeks after the cross-over) a third 24h IOP, BP and HR assessment was performed.

Despite there was no washout between the two treatment phases in the crossover design, the treatment duration in each phase was 8 weeks and allowed to remove the potential effect of the previous treatment (either timolol 0.5% or bimatoprost 0.01%) on the IOP of the second phase. This is based on the knowledge of the washout time for the study drugs which is known to be 4 weeks for timolol and 4–6 weeks for bimatoprost [6].

At baseline, week 8 and week 16, patients were admitted and IOP was measured by applanation tonometry at 6 time points over the 24h (in the sitting position by Goldmann applanation tonometry at 8:00, 12:00, 16:00 and 20:00 o’clock, and in the supine position by Perkins applanation tonometry at 00:00 and 04:00 o’clock). Two additional safety visits were performed at week 4 and 12 and during these visits IOP was measured in the sitting position by Goldmann applanation tonometry at 12.00 o’clock.

The mean of two readings or the median of three readings in case of differences >2 mmHg was reported for the analysis and the same calibrated Goldmann applanation tonometer was used by the same trained investigator in each center throughout the study.

At baseline visit patients’ ophthalmic and systemic history was recorded and gonioscopy, pachymetry, and standard automated acromatic perimetry, using the Humphrey 24–2 SITA Standard program, were performed. In addition to the IOP measurements a complete ophthalmological evaluation was performed at baseline and at each follow-up visits including best corrected visual acuity measurement, slit lamp examination, ophthalmoscopy and adverse events recording. At baseline and at week 8 and 16 systolic and diastolic BP and HR have been measured at each of the 6 time points of the 24h curve before the IOP measurements by the same trained investigator. Heart rate was measured manually at the radial artery by counting number of beats in 1 minute and blood pressure was measured manually by sphygmomanometry and it was reported as the mean value of three repeated measurement taken at each timepoint.

Additionally patients were asked to give specific answers to the following 3 questions at each follow-up visits: 1) did you notice any redness in your eyes since last visit? 2) did you experience any dry eye or foreign body sensation since last visit? 3) did you experience any eye burning sensation since last visit?

### Study medications

Two masked sets of study medications have been prepared, one set containing bimatoprost 0.01% for the evening administration and placebo for the morning administration and one set containing two bottles of timolol 0.5%, one for the morning and one for the evening administration. Masking, randomization list generation, labelling, blinding, packaging, and shipping of trial medications was handled by Pierrel Research IMP s.r.l..

At baseline, after randomization, 1 masked box containing 3 sets of study medications corresponding to treatment-phase 1 was dispensed to each randomized patient. At the week 8 visit the medications were collected from the patients and a second box containing 3 sets of study medications corresponding of treatment-phase 2 was dispensed to each patient.

During the admissions at baseline and at the week 8 and 16 visits the masked study drugs were administered according to the schedule by the study investigators.

### Study endpoints

The primary endpoint was the mean 24h IOP after 8 weeks of treatment and the primary comparison was the mean 24h IOP between the two active treatments. The secondary endpoints were: the mean IOP, BP and HR at each of the 6 timepoints of the 24h curve, the mean day (average of the 8:00, 12:00, 16:00 and 20:00 o’clock timepoints) and night (average of the 00:00 and 04:00 o’clock timepoints) IOP, BP and HR, the mean 24h diastolic and systolic ocular perfusion pressures (OPP, calculated as the difference between either the mean 24h systolic or the mean 24h diastolic blood pressure and the mean 24h IOP) and the incidence of adverse events. The secondary comparisons included the comparison between the active treatments and between each treatment and the baseline of: the mean timepoint IOP, the mean day and mean night IOP, the mean BP and HR, the mean diastolic and systolic OPP and the adverse events. Additionally the mean 24h IOP following each treatment in the two arms of the crossover design were compared between and within treatment phases.

The ocular perfusion pressure was calculated as the difference between either the mean 24h systolic or the mean 24h diastolic blood pressure and the mean 24h IOP.

### Statistical analysis

If only one eye was eligible as trial eye, that eye was included. If both eyes were eligible only the eye with highest baseline mean 24h IOP was included in the statistical analysis.

Only patients completing the baseline, 8 weeks and 16 weeks visits (24h IOP assessment visits) were included in the efficacy analysis. All randomized patients taking any amount of the study medication were included in the safety analysis.

Data have been described as mean and 95% confidence interval (CI) for continuous variables and frequencies for categorical variables. IOP data have been analysed by treatment and not by group, thus pooling data obtained from the same treatment irrespectively of the treatment sequence. A mixed effect model with time and drug as fixed effects and patients as random effect was used to test the null hypotesis that the mean 24h IOP between treatments is not different. The treatment sequence (either first bimatoprost 0.01% + placebo or first timolol 0.5% twice daily) was included in the model as fixed effect to test the interaction between drug and treatment sequence and to explore the presence of potential carry-over effects. Post-hoc paired comparisons have been performed by paired t-test or Wilcoxon sign rank test after normality check performed by Shapiro-Wilk test.

Categorical variables such as proportions and safety variables have been analyzed using the McNemar test. Statistical significance was set at the 5% level

We established a minimum sample of 29 participants based on the primary outcome (the comparison of the mean 24h IOP after 8 weeks of treatment pooling data from the same treatment in the crossover design) to have a 80% probability to detect a difference between treatments at a two-sided 0.05 significance level if the true difference between treatments is 1 mmHg. This is based on the assumption that the within-patient standard deviation of the response variable is 2 mmHg.

## Results

**Fig 1:**
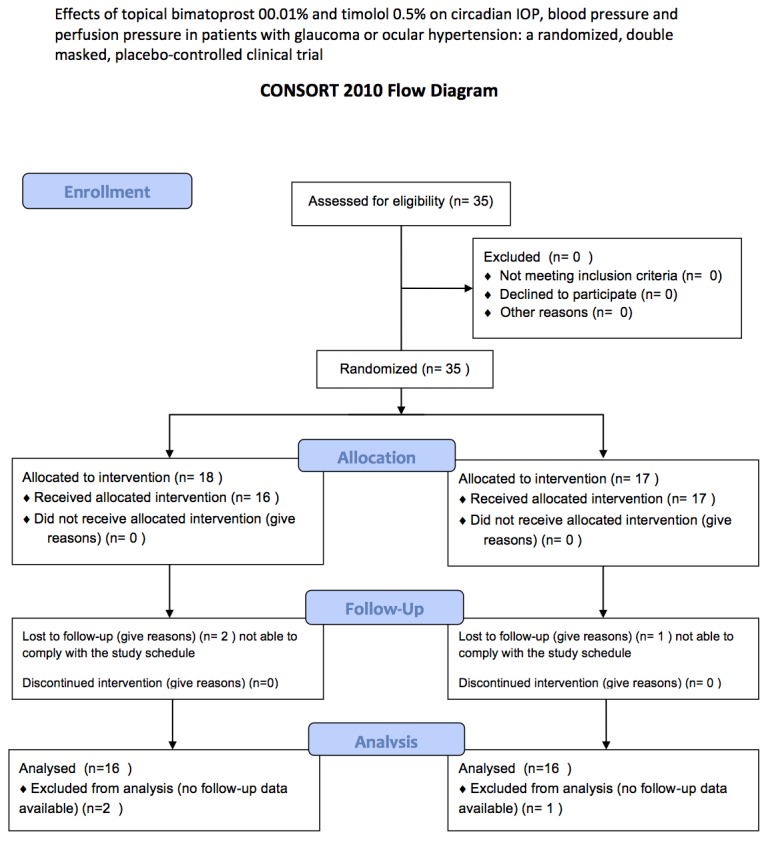
Study flow diagram.

A total of 35 patients were enrolled in this study between May and August 2012 and 32 were included in the efficacy and safety analysis (24 glaucomatous, 8 ocular hypertensive, mean age 61.0±11 years, 17 females and 15 males). Three patients were lost to follow-up after randomization without performing any follow-up visit and were excluded from the analysis (Fig 1). All enrolled patients had both eyes eligible for the study and received bilateral treatment but only data from the eye with the highest IOP at baseline were considered for the analysis.

Mean 24h IOP at baseline was 20.3 mmHg (95%CI 19.0 to 21.6) and after 8 weeks of treatment it was statistically significantly reduced by both treatments (15.7 mmHg, 95%CI 14.8 to 16.7, mean change from baseline -4.5 mmHg, 95%CI -3.6 to -5.4, p<0.0001 with bimatoprost 0.01% plus placebo; 16.8 mmHg, 95%CI 15.8 to 17.8, mean change from baseline -3.5 mmHg, 95%CI -2.6 to -4.4, with timolol 0.5% bid, p<0.0001).

Mean 24h IOP was statistically significantly lower after 8 weeks of treatment with bimatoprost 0.01% plus placebo than after 8 weeks of treatment with timolol 0.5% bid (mean difference between drugs -1.04, 95%CI -0.35 to -1.72, p = 0.0003). The mixed effects model confirmed that the mean 24h IOP changes from baseline were statistically significantly influenced by type of topical drug (p = 0.0041) with no statistically significant interaction between drug and treatment sequence (either first timolol 0.5% and second bimatoprost 0.01% or first bimatoprost0.01% and second timolol 0.5%) indicating no carry-over effects between drugs in the cross-over design (p = 0.97).

The paired comparisons of the mean 24h IOP in each arm of the crossover design confirmed that the mean 24h IOP during timolol 0.5% treatment was not different when timolol was used as first (16.5±2.5 mmHg) or as second drug (17.1±2.1 mmHg, p = 0.51) in the crossover design. Similarly the mean 24h IOP during bimatoprost 0.01% treatment was not different when bimatoprost was used as first (15.8±2.2 mmHg) or as second drug (15.7±2.6 mmHg, p = 0.9). Moreover the mean 24h IOP was statistically lower during bimatoprost 0.01% than during timolol 0.5% in both study arms so either when bimatoprost 0.01% was used as first drug (15.8±2.2 vs 17.1±2.1, p = 0.0008) and when it was used as second drug in the crossover design (15.7±2.6 vs 16.5±2.5, p = 0.013). This data also confirm the absence of any carry-over effects of the study drugs between the two study phases of the crossover design.

Mean day IOP (calculated as the mean of the IOP at the 8:00, 12:00, 16:00 and 20:00 o’clock timepoints) was statistically significantly reduced compared to baseline by both bimatoprost 0.01% plus placebo (-5.4 mmHg, 95% CI -4.4 to -6.4, p<0.0001) and by timolol 0.5% bid (-4.4 mmHg, 95% CI -3.5 to -5.3 p<0.0001).

Mean night IOP (calculated as the mean of the IOP at the 00:00 and 04:00 o’clock timepoints) was reduced by -2.3 mmHg (95%CI -1.2 to -3.4, p = 0.0002) compared to baseline by bimatoprost 0.01% plus placebo and by -1.1 mmHg (95%CI 0.0 to -2.3, p = 0.06) by timolol 0.5% bid. Full details are reported in Table 1.

**Table 1:** Comparison of mean intraocular pressure (mmHg) at baseline and after treatment with bimatoprost 0.01% once at night and after timolol 0.5% bid.

Measurement	Baseline	Timolol 0.5% bid	Mean difference from baseline	p value	Bimatoprost 0.01% plus placebo	Mean difference from baseline	p value	Mean difference between treatments	p value
	Mean (95% CI)	Mean (95% CI)	Mean (95% CI)	-	Mean (95% CI)	Mean (95% CI)	-	Mean (95% CI)	-
**Mean 24h IOP**	20.3 (19.0 to 21.6)	16.8 (15.8 to 17.8)	-3.5 (-2.6 to -4.4)	<0.0001	15.7 (14.8 to 16.7)	-4.5 (-3.6 to -5.4)	<0.0001	-1.0 (-0.3 to -1.7)	0.0003
**Mean Day IOP**	20.9 (19.6 to 22.3)	16.5 (15.5 to 17.5)	-4.4 (-3.5 to -5.3)	<0.0001	15.5 (14.6 to 16.4)	-5.4 (-4.4 to -6.4)	<0.0001	-1.0 (-0.3 to -1.6)	0.0007
**Mean Night IOP**	18.6 (17.2 to 20.0)	17.5 (16.1 to 18.8)	-1.1 (0.0 to -2.3)	0.056	16.3 (15.2 to 17.5)	-2.3 (-1.2 to -3.4)	0.0002	-1.1 (-0.2 to 2.1)	0.0019
**8:00**	22.5 (20.6 to 24.4)	17.5 (16.3 to 18.6)	-5.0 (-3.2 to -6.8)	<0.0001	15.9 (14.9 to 16.9)	-6.6 (-4.8 to -8.3)	<0.0001	-1.5 (-0.5 to -2.5)	0.0014
**12:00**	20.8 (19.0 to 22.2)	16.2 (15.1 to 17.4)	-4.5 (-3.2 to -5.9)	<0.0001	15.4 (14.3 to 16.5)	-5.4 (-4.1 to -6.8)	<0.0001	-0.9 (-0.1 to -1.7)	0.045
**16:00**	21.1 (19.5 to 22.8)	16.1 (14.9 to 17.4)	-5.0 (-3.9 to -6.1)	<0.0001	14.9 (13.8 to 15.9)	-6.3 (-4.8 to -7.7)	<0.0001	-1.3 (-0.2 to -2.4)	0.03
**20:00**	19.3 (18.0 to 20.5)	16.0 (14.8 to 17.2)	-3.3 (-2.1 to -4.5)	<0.0001	15.6 (14.4 to 16.8)	-3.7 (-2.4 to -4.9)	<0.0001	-0.4 (-0.7 to -1.6)	0.56
**00:00**	18.2 (16.8 to 19.5)	17.3 (15.9 to 18.7)	-0.9 (+0.6 to -2.1)	0.23	16.1 (14.9 to 17.4)	-2 (-0.65 to -3.4)	0.0034	-1.2 (-0.2 to -2.1)	0.0069
**04:00**	19.1 (17.3 to 20.8)	17.6 (16.2 to 19.1)	-1.4 (0.0 to -2.8)	0.092	16.6 (15.3 to 17.8)	-2.5 (-1.0 to -4.0)	0.0014	-1.0 (0.0 to -2.2)	0.012

IOP = Intraocular pressure; CI = confidence interval; bid = bis in die. Mean day IOP = mean of 08:00, 1200, 16:00 and 20:00 o’clock timeponts; Mean night IOP = mean of 00:00 and 04:00 o’clock timepoints.

**Fig 2:**
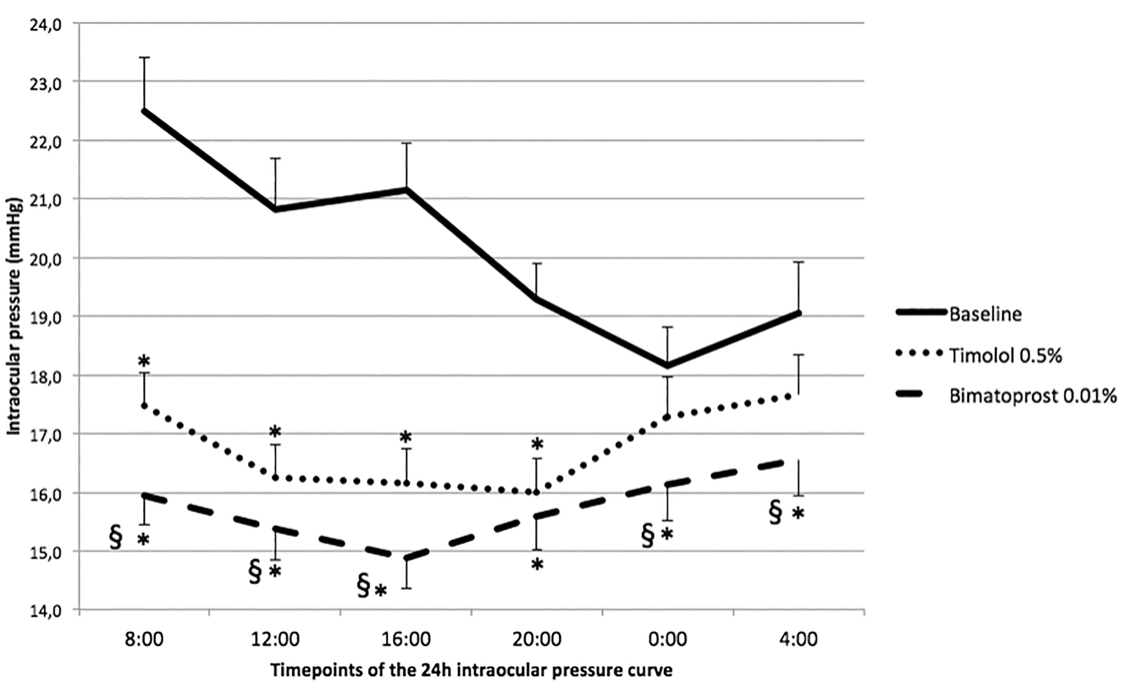
Intraocular pressure at each time point of the daily curve. Error bars represents standard error; * = p<5% for timepoint comparison versus baseline; § = p<5% for timepoint comparison between timolol 0.5% bid and bimatoprost 0.01% plus placebo.

The mean IOP at all individual timepoints of the 24h curve after 8 weeks of treatment with bimatoprost 0.01% plus placebo was significantly lower than the corresponding timepoints at baseline (full details are given in Table 1). The mean IOP measured at the day timepoints of the 24h curve (8:00, 12:00, 16:00, 20:00) after 8 weeks of treatment with timolol 0.5% bid was significantly lower compared to the corresponding timepoints at baseline. The mean IOP at the 00.00 and 04.00 timepoints (night timepoints) were not statistically significantly lower than the baseline values (full details in Table 1 and Fig 2).

**Fig 3:**
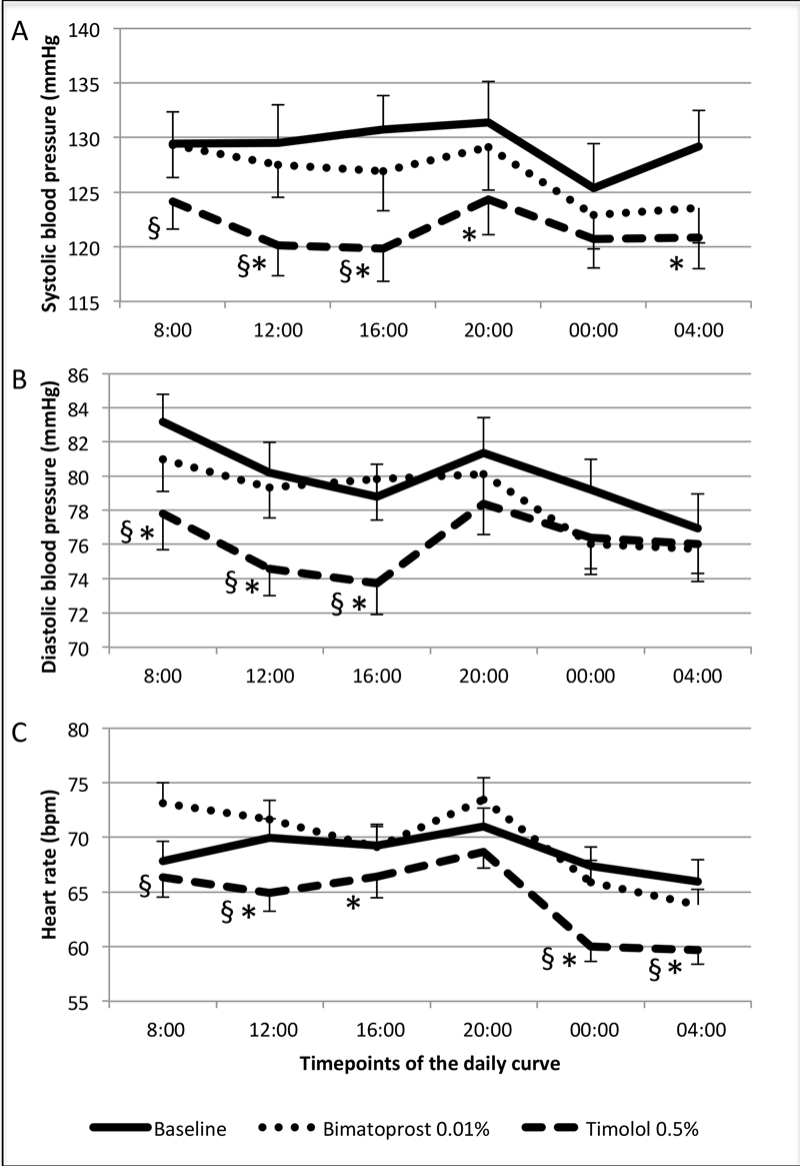
Systolic (A) and diastolic (B) blood pressure and heart rate (C) at each timepoint of the 24h curve at baseline (continuous line), during timolol 0.5% bid (dashed line) and during bimatoprost 0.01% plus placebo treatment (dotted line). Bpm = beats per minute. Error bars represents standard error; * = p<5% for timepoint comparison versus baseline; § = p<5% for timepoint comparison between timolol 0.5% bid and bimatoprost 0.01% plus placebo.

**Fig 4:**
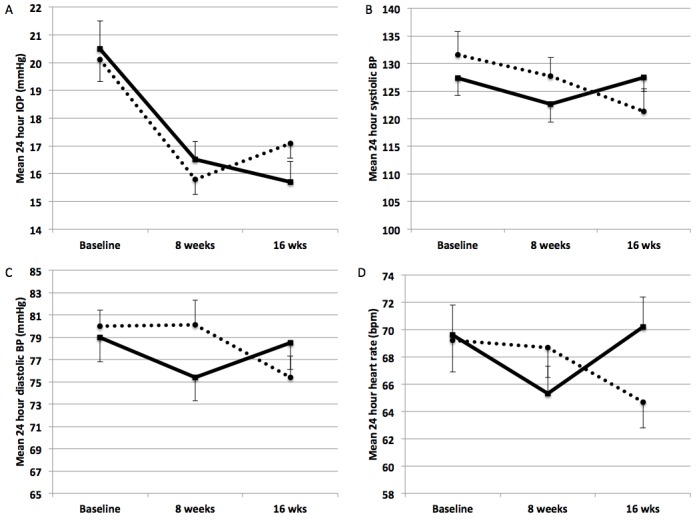
Display of the mean 24 hour IOP (A), systolic BP (B), diastolic BP (C) and heart rate (D) of the two arms of the study at baseline, at 8 weeks before the crossover and at 16 weeks after the crossover. The dotted line indicates the study arm that received bimatoprost 0.01% plus placebo as first treatment and timolol 0.5% bid as second treatment. The continous line indicates the study arm that received timolol 0.5% bid as first treatment and bimatoprost 0.01% plus placebo as second treatment. IOP = intraocular pressure, BP = blood pressure.

After 8 weeks of treatment with timolol 0.5% bid systolic BP was significantly lower compared to baseline at all but one (00.00 o’clock) timepoints of the 24h curve. After 8 weeks of treatment with bimatoprost 0.01% systolic BP was not different from the baseline values at all timepoints of the 24h curve (Fig 3A). After 8 weeks of treatment with timolol 0.5% bid diastolic BP was significantly lower compared to baseline at the 8.00, 12:00 and 16:00 o’clock timepoints. After 8 weeks of treatment with bimatoprost 0.01% diastolic BP was not different compared to the baseline at all timepoints (Fig 3B). Heart rate was significantly lower compared to baseline at 12:00, 16:00, 00:00 and 04:00 after timolol 0.5% bid treatment and was unchanged at all timepoints compared to baseline after bimatoprost 0.01% treatment (Fig 3C). The effects of the 2 treatment sequences on IOP, BP and HR are also displayed separately in Fig 4.

Mean 24h systolic OPP was 109.2±15.7 mmHg at baseline and was significantly lower after 8 weeks of treatment with timolol 0.5% bid (105.2±11.4, p = 0.024) while it was unchanged compared to baseline after treatment with bimatoprost 0.01% (110.9±14.5 mmHg, p = 0.41). Mean 24h systolic OPP was lower after 8 weeks treatment with timolol 0.5% bid than after treatment with bimatoprost 0.01% plus placebo (105.2±11.4 vs 110.9±14.5 mmHg, mean difference -5.7 mmHg (95%CI -2.99/-8.4 mmHg), p = 0.0002).

Mean 24h diastolic OPP was significantly higher than baseline after 8 weeks treatment with bimatoprost 0.01% (63.1±9.1 mmHg vs 59.5±7.7 mmHg, p = 0.0013) while it was similar to the baseline value after 8 weeks of treatment with timolol 0.5% bid (59.8±7.3 mmHg vs 59.5±7.7 mmHg, p = 0.72). The mean 24h diastolic OPP was higher after 8 weeks treatment with bimatoprost 0.01% than after treatment with timolol 0.5% bid (63.1±9.1 vs 59.8±7.3 mmHg, men difference 3.3 mmHg (95%CI 1.5/5.2 mmHg) p = 0.0009).

Concerning local tolerability burning was reported by 1 patient (3.1%) during timolol 0.5% treatment and by 2 patients (6.2%, p = 1.0) during bimatoprost 0.01% treatment. Conjunctival hyperemia was reported by 2 patients (6.2%) during timolol 0.5% treatment and by 2 patients (6.2%, p = 1.0) during bimatoprost 0.01% treatment. One patient reported episodes of extra systole during timolol 0.5% treatment and they were judged as not related with the study drugs. No serious adverse events occurred during the study.

## Discussion

In this study the 24h ocular hypotensive efficacy and the effects on BP and HR of bimatoprost 0.01% administered once at night were investigated and compared to those of timolol 0.5% administered twice daily. According to our results both bimatoprost and timolol are effective in reducing the mean 24h IOP in glaucomatous and ocular hypertensive subjects from an average untreated baseline IOP of 20.3 mmHg. Nevertheless the mean 24h IOP at the end of the follow-up was found to be significantly lower with bimatoprost 0.01% than with timolol 0.5%. Moreover According to our data, bimatoprost 0.01% and timolol 0.5% exerted different effects on the 24h IOP profiles. In fact, when patients were treated with bimatoprost 0.01%, the IOP was found to be significantly reduced at each individual time-point of the 24h curve compared to the corresponding baseline untreated timepoint and this was true for both the day and for the night time-points. Differently when patients were on timolol 0.5% IOP was found to be lower than baseline at all day time-points (08:00, 12:00, 16:00, 20:00 o’clock) but not at 00.00 and 04:00 o’clock (night timepoints). This finding is in agreement with previous works by Orzalesi et al. [14] who compared the 24h IOP reduction induced by timolol 0.5% and the hypotensive lipid latanoprost 0.005%, in glaucoma or ocular hypertension patients and found that while latanoprost seemed to lead to a fairly uniform circadian reduction in IOP timolol seemed to be less effective during the nighttime hours. Our results are also in agreement with those by Quaranta et al. [15] who found that timolol induced a greater reduction in IOP during the day (8 AM to 8 PM) and a less reduction during the night and are in accordance also with Konstas et al. [16].

Liu and co-authors [17] investigated the nocturnal effects of once-daily timolol and latanoprost on IOP in patients with ocular hypertension or glaucoma and found that in the nocturnal period, IOP with timolol treatment was not different from the IOP with no medication and was significantly higher than IOP with the latanoprost treatment.

More recently the same group published the results of a prospective, open-label experimental 24h investigation of the effects of bimatoprost 0.01% monotherapy on IOP and ocular perfusion pressure and reported that the diurnal and nocturnal IOP was significantly lower, and the ocular perfusion pressure was significantly higher, with bimatoprost 0.01% treatment compared to an untreated baseline [18].

Our findings are likely to be related to different effects on acqueus humor dynamics exerted by bimatoprost and timolol over the 24h as recently reported by Gulati and co-workers who explored the aqueous humor dynamics in a cross-over, randomized, double masked clinical study in patients treated with different ocular hypotensive medications. The authors found that during the night-time aqueous inflow is physiologically reduced in untreated eyes by 47% and that topical treatment with timolol 0.5% is substantially ineffective in further reducing the inflow during the night hours with consequent weak effect on IOP [19]. Interestingly in the same study it was found that prostaglandin analogues (latanoprost) significantly reduced the IOP during both day and night but the reduction at night was less than the reduction obtained during the day and this finding was associated with a reduced effect on uveoscleral outflow during the night hours. This finding is in agreement with the behaviour of IOP observed in the present study with bimatoprost 0.01% which was associated with a significant reduction of IOP throughout the 24 hours that appeared less pronounced during the night hours.

Our results are not in agreement with the results of a systematic review and meta-analysis by Lee and co-workers [20]. The authors evaluated the night-time IOP and BP response to timolol treatment in patients with ocular hypertension or primary open-angle glaucoma from 9 previously published articles involving overall 340 patients. The results suggest that topical timolol therapy provides an ocular hypotensive effect over the 24-hour curve, including the nighttime hours, with small reductions in the systolic and diastolic pressures and no alterations of the ocular perfusion pressure over 24 hours. The disagreement with the results of the present study could be due to several factors mainly related to differences in the sample populations and in the study methodology. In fact in our study IOP was measured in the supine position at the night time-points while in 8 out of the 9 articles included in the meta-analysis the night IOP was measured only in the sitting position.

In addition to the investigation of IOP, in this study we explored the 24h behaviour of the systolic and diastolic blood pressure and heart rate, and we found significant differences between he two treatment periods.

In fact, systolic, diastolic blood pressure and heart rate values were found not different compared to the untreated baseline values during bimatoprost 0.01% while they showed significant changes during treatment with the timolol 0.5%. Specifically the systolic blood pressure was found to be reduced at the night time-points (00:00 and 04:00) and at 3 out of 5 day timepoints (12:00, 16:00, 20:00), while the diastolic blood pressure showed significant changes mainly during the day with a significant dip at 08:00, 12:00 and 16:00 o’clock (Fig 3). Also the heart rate was found significantly reduced by timolol 0.5% at 5 out of 7 timepoints of the 24h curve with mean nocturnal values reaching 60 bpm at 00:00 and 04:00. These findings are in agreement with previous reports that showed significant changes in the cardiovascular parameters during treatment with topical beta-blockers. Netland et al. showed in a randomized, double-masked investigation of the 24h behaviour of cardiovascular parameters under topical treatment with different beta-blockers that timolol causes a significant reduction in heart rate from midnight to 04:00 o’clock and that 18.4% of patients became clinically significantly bradycardic (heart rate <60 bpm) during topical timolol treatment [8].

Despite these findings confirm that the well known effects of beta blockers on blood pressure and heart rate when administered systemically may be exerted also when they are administered topically in the form of eyedrops the clinical significance of the observed changes remains to be ascertained. Hayreh et al. investigated the effect of topical beta-blocker eyedrops on nocturnal arterial hypotension and heart rate and on visual field deterioration by prospectively following 161 patients with glaucomatous optic neuropathy and they found that the use of beta-blockers eyedrops is associated with nocturnal hypotension and reduced heart rate and that these effects may be a potential risk factor for disease progression and visual field deterioration in vulnerable individuals [7]. Nevertheless despite the pathogenetic mechanisms underlying this relationship have been not yet fully characterized Graham and Drance in their review on the role of nocturnal hypotension in glaucoma concluded that there is enough evidence to state that nocturnal hypotension may play a role in the progression of glaucomatous damage probably via reduced perfusion of the optic nerve head [21].

In our study we calculated the mean 24h systolic and diastolic ocular perfusion pressure and we found that while the diastolic was increased during bimatoprost 0.01% treatement (as the result of a significant reduction of the IOP associated with no effects on blood pressure), the systolic was found significantly reduced during timolol treatment (as the result of a lower effect on the IOP associated with a reduction of the systolic blood pressure). Nevertheless it has to be highlighted that ocular perfusion pressure is an arithmetically calculated vascular measure whose clinical role is still under debate. Bonomi et al. in the Egna-Neumarkt Study found that a lower diastolic perfusion pressure was associated with a marked, progressive increase in the frequency of glaucoma [22]. More recently Caprioli et al., in their review about blood pressure, perfusion pressure and glaucoma, concluded that low perfusion pressure and blood pressure are associated with glaucoma despite there is still no evidence to support the value of correcting low blood pressure as therapy for glaucoma mainly for the lack of crucial information about the microvascular beds in which perfusion is important in glaucoma, and for the cardiovascular safety concerns associated with treatments designed to increase ocular perfusion pressure and blood flow by increasing blood pressure, especially in elderly patients [23]. In this context however, the results of the present study may help the clinician in personalizing the topical treatment according to the individual patient’s glaucoma and cardiovascular characteristics.

Moreover the findings observed in our study of reduced blood pressure, heart rate and ocular perfusion pressure with timolol 0.5% administered twice daily associated with a good daily effect on IOP but a weak effect on IOP during the night-time might suggest that timolol 0.5% administered only once in the morning might have a similar 24h effect on IOP with reduced impact on cardiovascular parameters. Nevertheless for a evidence-based once daily use of timolol 0.5% future prospective specifically designed clinical trials are required.

Finally in the present trial both drugs under investigation showed a very good tolerability profile with very low incidence of side effects. This finding is of particular interest considering that patients were actively asked about the occurrence of specific side effects such as burning, stinging, foreign body sensation or conjunctival hyperemia and despite the specific questions the positive answers were very low for both timolol and bimatoprost 0.01%.

One potential bias of crossover studies is the presence of potential carry-over effects of the first treatment on the second treatment. We tested and excluded the presence of any carry-over effect in the present study. The absence of carry-over effects is also supported by the finding of similar mean 24h IOP in patients who received timolol 0.5% as first drug compared to patients who received timolol 0.5% as a second drug (after bimatoprost). Similarly the mean 24h IOP was not different in patients treated with bimatoprost 0.01% as a first drug compared to patients treated with bimatoprost 0.01% as second drug. Additionally the mean 24h IOP was statistically lower during bimatoprost 0.01% than during timolol 0.5% in both study arms so either when bimatoprost 0.01% was used as first drug and when it was used as second drug in the crossover design.

Among the limitations of the present trial it has to be highlighted that that the results refer to the short term and might require confirmation in the long term and that only primary open angle glaucoma patients and ocular hypertension subjects have been enrolled and that the study results might not be generalisable to angle closure or secondary glaucomas. Moreover it has to be highlighted that the present study was not designed to assess visual function outcomes and no information can be directly derived about the relative effectiveness of the different drugs in preventing the progression of visual field loss. Nevertheless large randomized clinical trials, such as the EMGT and the CGS, have reported a significant reduction of the risk of glaucoma progression (-11% to -19%) associated to each mmHg of IOP reduction obtained by treatment, thus supporting the clinical relevance of differences of effectiveness between drugs starting from 1 mmHg [2,4].

In conclusion, our results suggest that both bimatoprost 0.01% administered once at night and timolol 0.5% administered twice daily are effective in reducing the mean 24h IOP with timolol being less effective during the night hours. The observed effects of timolol on the systolic and diastolic blood pressure and heart rate might be clinically irrelevant in healthy individuals but should be considered in the process of choosing the right treatment for the individual glaucoma and ocular hypertensive patient also considering the use of lower concentrations of the active drug.

## Supporting Information

S1 CONSORT Checklist (DOC)

S1 File Study protocol in Italian. (PDF)

S2 File Study Protocol in English. (PDF)
