# Supplementary material for: Effects of Topical Bimatoprost 0.01% and Timolol 0.5% on Circadian IOP, Blood Pressure and Perfusion Pressure in Patients with Glaucoma or Ocular Hypertension: A Randomized, Double Masked, Placebo-Controlled Clinical Trial
Source: PLoS One. 2015 Oct 20;10(10):e0140601. doi: 10.1371/journal.pone.0140601 (PMC4615626; doi:10.1371/journal.pone.0140601)
Supplement: S1 File — (PDF) [file pone.0140601.s002.pdf]

Confronto tra gli effetti del Bimatoprost 0.01% e Timololo 0.5% sulla pressione intraoculare ed arteriosa circadiane

EudraCT number 2010-024272-26

Sponsor dello studio

IRCCS G.B.Bietti Eye Foundation
Via Livenza 3
00198, Roma, Italia

Investigatore principale

Prof. Marco Centofanti
Glaucoma Division
IRCCS G.B.Bietti Eye Foundation
Via Livenza 3
00198, Roma, Italia

Centri partecipanti

IRCCS G.B.Bietti Eye Foundation
Via Livenza 3
00198, Roma, Italia
Investigator: Dr. Francesco Oddone

INTRODUZIONE

Il Bimatoprost 0.03% (bimatoprst 0.3) è un potente analogo prostamidico capace di ridurre la pressione intraoculare (IOP) del 33% (-35% a -31%), durante il giorno e del -28% (-29% a -27%) nelle ore notturne ed è quindi molto efficace nel controllare la pressione intraoculare nel corso delle 24 ore.

E' stata recentemente approvata, come terapia di prima linea, una nuova formulazione di

Bimatoprost 0.01% (bimatopsot 0.1) capace di ridurre la pressione intraoculare sia nei pazienti con glaucoma ad angolo aperto sia nei pazienti con ipertensione oculare, ma non è stata ancora dimostrata la sua efficacia e la sua sicurezza sulla IOP nelle 24 ore attraverso uno studio di fase IV.
La nuova formulazione di bimatoprost contiene una concentrazione più bassa di principio attivo , questa caratteristica consente a tale formulazione di ridurre gli effetti collaterali locali, come ad esempio l'iperemia congiuntivale. La riduzione degli effetti collaterali è di estrema importanza se si considera che il glaucoma è una patologia cronica, così come cronica è la sua terapia. È stato recentemente dimostrato che il bimatoprost 0.1 possiede una tollerabilità oculare superiore al bimatoprost 0.3 e le due formulazioni sembrano avere un'efficacia equiparabile. Tuttavia non si conoscono gli effetti di questa nuova formulazione sulla curva tonometrica delle 24 ore. Sarebbe quindi utile indagare, se il bimatoprost 0.1, pur avendo una concentrazione inferiore rispetto al bimatoprost0.3, l'effetto ipotonizzante riesce a perdurare durante le 24 ore. Infatti in alcune forme di glaucoma, soprattutto in quelle progressive, è importante sia che la pressione intraoculare sia bassa sia che non ci siano grandi variazioni nell'arco della giornata.
L'obiettivo principale di questo studio è valutare l'efficacia e la sicurezza della nuova formulazione bimatoprost 0.1

DISEGNO DELLO STUDIO

Studio prospettico, cross-over, randomizzato, in doppio cieco con controllo attivo.

POPOLAZIONE IN STUDIO

Soggetti con POAG o OHT di nuova diagnosi non in terapia ipotonizzante oculare e con qualsiasi valore tonometrico o pazienti con POAG o con OHT già trattati in entrambi gli occhi con farmaci ipotensivi oculari in monoterapia e con una IOP <22 mmHg.

MATERIALI E METODI

Potranno essere reclutati nello studio sia pazienti con ipertensione oculare(OHT) o pazienti con glaucoma ad angolo aperto (OAG) di nuova diagnosi, non in terapia ipotensiva oculare sia pazienti in terapia ipotonizzante oculare. I pazienti non in terapia devono essere pazienti in cui la pressione intraoculare possa essere controllata da una monoterapia a giudizio dello sperimentatore. I pazienti già in terapia devono avere una pressione intraoculare inferiore a 22 mmHg in

monoterapia. I pazienti in terapia ipotonizzante oculare verranno sottoposti alla curva tonometrica nictemerale basale, dopo un adeguato periodo di sospensione dalla terapia ipotonizzante oculare al fine di valutare la pressione oculare basale e valutare l'effettiva l'efficacia del farmaco in studio

Criteri d'inclusione

- Firma del consenso informato
- Età >18 anni
- Diagnosi di POAG o OHT. La definizione di POAG o di OHT è basata sulle linee guida dell' "European Glaucoma Society"
- Pazienti di nuova diagnosi (OHT e POAG) e mai trattati con farmaci ipotensivi oculari con qualsiasi valore tonometrico e pazienti con OHT e POAG in terapia con farmaci ipotensivi oculari in monoterapia e con IOP< 22mmHg alla visita di screening.

Criteri d'esclusione

- Inabilità a firmare il Consenso informato
- Condizioni oculari che posso interferire con i risultati dello studio
- Angolo stretto o storia di attacchi acuti di glaucoma
- Infezioni o infiammazioni oculari nei 3 mesi precedenti alla visita di screening
- Chirurgia oculare, trabeculoplastica o argon laser negli ultimi tre mesi.
- Storia di Ipersensibilità al benzalconio cloruro o ad altri componenti contenuti nella formulazione del farmaco in studio
- Uso di farmaci topici che possano interferire con il farmaco in studio
- Cause di aumento secondario delle pressione intraoculare
- Controindicazioni sistemiche all'utilizzo dei farmaci in studio:
    - Ipersensibilità al principio attivo o agli eccipienti contenuti nel farmaco.
    - Patologie bronchiali (asma bronchiale, patologie ostruttive delle vie aeree)

- Patologie cardiache (bradicardia sinusale, blocco atrioventricolare di secondo o terzo grado, insufficienza cardiaca, shock cardiovascolare, etc etc)
- Rinite allergica severa o iperattività bronchiale, distrofie corneali, ipersensibilità ad altri beta-bloccanti

- Altre condizioni o sintomi che a giudizio del ricercatore precludano l'arruolamento nello studio
- Pazienti sottoposti a chirurgia refrattiva
- Donne in gravidanza o potenzialmente fertili (possono essere reclutate donne in menopausa spontanea da almeno un anno o con amenorrea da almeno sei mesi con livelli di FSH maggiori di 40mUI/ml o che siano stati sottoposte a ovariectomia con o senza isterectomia o che usino un adeguato metodo anticoncezionale)
- Incapacità ad aderire al regime di trattamento o alle procedure previste dallo studio
- Partecipazione ad altri studi clinici nei tre mesi precedenti alla visita di screening

RANDOMIZZAZIONE E SCHEMA DELLO STUDIO

Tutti i pazienti dopo la curva tonometrica basale verranno assegnati in maniera randomizzata ad un gruppo di trattamento.

I trattamenti possibili saranno due: i pazienti assumeranno o il Bimatoprost 0.01%(Bimatoprost 0.1) una volta alla sera ed il placebo al mattino, o il Timololo 0,5% due volte al giorno.

Dopo 8 settimane, ai pazienti verrà effettuata una seconda curva tonometrica delle 24 ore e, subito dopo, i pazienti passeranno all'altro regime terapeutico dello studio (i pazienti in terapia con Bimatoprost 0,01% passeranno al Timololo0,5% e viceversa) e verranno seguiti per altre 8 settimane.

Alla fine del follow-up (16 settimane rispetto al basale e 8 settimane dopo il crossover) verrà effettuata una terza curva tonometrica nictemerale. (Vedere diagramma delle visite)

PERIODO DI SOSPENSIONE DELLA TERAPIA IPOTONIZZANTE OCULARE

La sospensione momentanea della terapia ipotonizzante oculare verrà iniziata alla visita di arruolamento solo ai pazienti con POAG o OHT che sono in terapia con un trattamento topico ipotensivo oculare, al fine di valutare la pressione basale.

Il tempo di sospensione varierà a seconda del trattamento in corso:

- 6 settimane per i derivati prostaglandinici (Latanoprost, Bimatoprost, Travoprost)
- 4 settimane per betabloccanti selettivi e non selettivi (Timololo, Levobunololo, Carteololo, Betaxololo)
- 4 settimane per gli α-agonisti (Apraclonidina, Brimonidina)
- 2 settimane per gli inibitori di anidride carbonica (Dorzolamide, Brinzolamide)
- 2 settimane per i miotici (Pilocarpina, Glamidolo, Aceclidina)

OBIETTIVI

Obiettivo primario: valutare l'efficacia del Bimatoprost 0.01% (somministrato una volta al giorno, alla sera) rispetto al Timololo 0.5% (somministrato due volte al giorno) nel ridurre la IOP durante le 24 ore.

Endpoint primario: confrontare la pressione intraoculare media dopo 8 settimane di trattamento con Bimatoprost 0.01% e Timololo 0.5%.

Endpoint secondari:

Valutare dopo 8 settimane di trattamento:

- I cambiamenti della IOP media durante le 24 ore rispetto alla visita basale.
- La IOP media ad ogni orario di misurazione durante la curva giornaliera.
- I cambiamenti della IOP media di ogni misurazione della curva giornaliera.
- La media della IOP giornaliera (8:00, 12:00, 16:00, 20:00) e media della IOP notturna (0:00, 4:00).
- La media della pressione arteriosa diastolica e sistolica e della frequenza cardiaca durante le 24 ore.

- La pressione di perfusione oculare sistolica e diastolica.
- La presenza di eventi avversi.
- Le risposte del questionario riguardo alla tollerabilità del farmaco

VISITE DELLO STUDIO E SCHEMA DEL TRATTAMENTO

Durante la **Visita di Arruolamento** verrà valutato se i pazienti sono idonei a partecipare allo studio.

Qualora eleggibili, dopo la firma del consenso informato, gli verrà chiesto di interrompere qualsiasi trattamento topico ipotensivo oculare.

La visita di base verrà programmata dopo un appropriato periodo di sospensione (vedi paragrafo periodo di sospensione della terapia ipotonizzante oculare).

Durante la **Visita Basale** ai pazienti verrà misurata la IOP alle 8:00, 12:00, 16:00, 20:00, 0:00, 4:00, 8:00.

Dopo l'ultima misurazione della IOP, i pazienti verranno randomizzati in 2 gruppi. Ad un gruppo verrà assegnato il placebo la mattina (8:00) e il Bimatoprost 0.01% la sera (20:00), ad un altro gruppo verrà assegnato il Timololo 0.5% due volte al giorno(8:00 – 20:00) .

Ad ogni paziente verrà distribuita una confezione in cui il nome del farmaco sarà mascherato. Sia il paziente sia il medico che lo segue durante le procedure del protocollo non conosceranno quale farmaco il paziente instilla durante quel periodo.

Dopo 4 settimane dalla randomizzazione ai pazienti verrà effettuata una **Visita di Sicurezza (Visita 1A)** dove verrà misurata la IOP alle 12:00, la pressione sanguigna e la frequenza cardiaca.

Dopo 8 settimane dalla randomizzazione ai pazienti **(Visita 2A)** verrà misurata la IOP negli stessi orari della visita basale, cioè alle 8:00, 12:00, 16:00, 20:00, 0:00, 4:00, 8:00.

Dopo l'ultima misurazione delle IOP ai pazienti verrà chiesto di sospendere il primo trattamento e gli verrà dato il farmaco per il secondo periodo di trattamento.

I pazienti che precedentemente instillavano il Bimatoprost 0.01% alla sera e placebo al mattino passeranno al Timololo 0.5% due volte al giorno(mattina e sera) e i pazienti che precedentemente sono stati trattati con il Timololo 0.5% passeranno al Bimatoprost 0.01% + Placebo.

Dopo 4 settimane dal crossover, verrà effettuata una **Visita Di Sicurezza (Visita 1B)** in cui verrà effettuata una misurazione della IOP alle ore 12:00, la misurazione della pressione sanguigna e della frequenza cardiaca.

Dopo 8 settimane dal crossover, i pazienti saranno sottoposti alla misurazione della IOP**(Visita 2B)**durante le 24 ore negli stessi orari della visita 2A e della visita basale (alle ore 8:00, 12:00, 16:00, 20:00, 0:00, 4:00, 8:00).

Nelle visite 2A e 2B il farmaco che il paziente deve assumere al mattino verrà somministrato dopo la misurazione delle ore 8.00 così come il farmaco che il paziente assume alla sera verrà instillato dopo la misurazione della IOP delle ore 20.00.La pressione arteriosa diastolica e sistolica e la frequenza cardiaca verranno monitorate prima di ogni misurazione della IOP della curva giornaliera, durante la visita di base,la visita 1°, la visita 2A , la visita 1B, la visita

SELEZIONE DELL'OCCHIO IN STUDIO

Se solo un occhio è idoneo allo studio, includeremo nel trattamento solo l'occhio eleggibile e questo sarà inserito nell'analisi statistica. Se sono idonei entrambi gli occhi, verranno trattati entrambi ma solo l'occhio con la media della IOP più alta alla visita basale verrà incluso nell'analisi statistica.

ESCLUSIONE DEI SOGGETTI

- La gravidanza è una causa di esclusione dallo studio.
- Un paziente verrà escluso dallo studio per motivi medici, a giudizio del ricercatore,
- Un paziente può decidere di essere escluso dallo studio perché lo desidera.
- Un paziente verrà escluso dallo studio nel caso in cui vi è un fallimento della terapia (IOP>24mmhg) dopo qualsiasi visita successiva alla randomizzazione.

Se un soggetto non ritorna nel giorno della visita programmata il ricercatore che lo segue deve sollecitarlo prontamente per fissargli un nuovo appuntamento ad una distanza massima di 7 giorni da quello stabilito in precedenza

FARMACI IN STUDIO E MASCHERAMENTO

I farmaci dello studio verranno acquistati dallo Sponsor (IRCCS Fondazione G.B.Bietti).

Mascheramento, randomizzazione (per centro), etichettatura, imballaggio e spedizione del farmaco in studio saranno gestiti da *PIERREL RESEARCH IMP S.R.L.Via Como 5, 22063 Cantù (CO), Italia.*

Verranno preparati due gruppi di farmaci mascherati. Un kit conterrà Bimatoprost 0,01% e placebo e un altro kit conterrà due flaconi di Timololo 0,5%, uno per la somministrazione della mattina e uno per la somministrazione serale.

Alla visita di baseline dopo la randomizzazione verrà distribuita ad ogni paziente una scatola con 3 serie di farmaci corrispondente al Trattamento-Fase1.

Alla visita 2A a ciascun paziente verrà consegnata una seconda scatola con un’altra serie di farmaci corrispondente al Trattamento–Fase 2.

MISURAZIONE DELLA IOP E VALUTAZIONE DELL’ EFFICACIA DEL TRATTAMENTO

La IOP verrà misurata alle 8:00, 12:00, 16:00, 20:00, 0:00, 4:00, 8:00 nella visita basale, nella visita 2A e nella visita 2B.

La IOP verrà misurata solo alle ore 12:00 nella visita 1A e 1B.

E’ consentita una deviazione di ≤15 minuti per ogni misurazione.

La IOP verrà misurata con il tonometro ad applanazione di Goldmann.

Il valore della IOP sarà il valore medio di 2 misurazioni o il valore della mediana di 3 misurazioni della IOP qualora esistesse una differenza > 2 mmHg tra le prime 2 misurazioni.

Deve essere utilizzato un solo tonometro di Goldmann calibrato, ed un solo ricercatore, dopo un adeguato training, potrà misurare la pressione dell’occhio durante tutto lo studio.

La durata del trattamento in ogni fase dello studio è di 8 settimane e tale periodo risulta sufficientemente lungo per evitare che l'effetto delle misurazioni durante le curve tonometriche sia influenzato dal effetto del trattamento precedente, infatti il periodo di sospensione per i betabloccanti topici è di 4 settimane e per le prostaglandine è di 6 settimane.

La valutazione della pressione intraoculare dopo 8 settimane dal cambio di terapia consentirà di evitare ogni effetto derivante dal trattamento precedente.

COMPLIANCE DEL TRATTAMENTO

I pazienti verranno istruiti dai ricercatori su come e quando instillare la terapia topica. Nel questionario del CRF verrà verificato se il paziente avrà effettuato correttamente la terapia.

TERAPIA CONCOMITANTE

Durante lo studio non può essere utilizzato, in entrambi gli occhi, nessun altro farmaco topico oftalmico e non può essere né iniziata, né modificata nessuna terapia sistemica che abbia effetti noti sulla IOP (ad esempio antagonisti beta-adrenergici, agonisti alfa-adrenergici, i calcio antagonisti, ACE-inibitori e antagonisti del recettore dell'angiotensina II).

Altre terapie considerate necessarie per il paziente, possono essere somministrate, a discrezione del ricercatore.

Tutte le terapie devono essere registrate sul Case Report Form (CRF). Nessun altro farmaco topico oculare può essere usato in concomitanza con il farmaco in studio. I pazienti non sono autorizzati a partecipare contemporaneamente ad altre sperimentazioni cliniche.

VALUTAZIONE DELLA SICUREZZA

Acuità visiva e Refrazione:

Durante la visita basale e durante le visite visita 2A e 2B verrà valutata l'acuità visiva (Snellen) con la migliore correzione e registrata sul CRF. Una riduzione clinicamente rilevante dell'acuità visiva deve essere riportata tra gli eventi avversi.

Annessi oculari e esame della lampada a fessura:

Esame della lampada a fessura e degli annessi oculari verrà effettuato durante tutte le viste dello studio. Verrà osservata la cute ed il bordo palpebrale superiore ed inferiore.

Bisognerà registrare e indicare il grado di severità nel caso in cui si osserverà la presenza di:

eventuali depositi di pigmento negli strati corneali endoteliali, anomalie sulla superficie palpebrale, della congiuntiva, del cristallino, della cornea, della camera anteriore, dell'iride.

La presenza di pseudofachia dovrà essere registrata.

La presenza di cellule in camera anteriore verrà "gradata" come lieve=1(3-5 cellule) moderata=2 (6-20 cellule) e severa= 3 (>20 cellule)

La presenza dell'iperemia sarà definita sia obiettivamente sia potrà essere riferita dal paziente.

Oftalmoscopia: Alla visita di base e alla visita 2B verrà effettuata l'oftalmoscopia per esaminare:

- il vitreo (se il vitreo presenta delle anomalie, segnalare il grado lieve=1, moderato=2 o severo=3).
- la retina (se vi è presenza di eventuali patologie a carico della retina (macula e periferia)segnalare il grado lieve=1, moderato=2 o severo=3).

  il disco ottico (se vi è presenza di eventuali patologie segnalare il grado lieve=1, moderato=2 o severo=3). Verrà registrato il cup/disc orizzontale e verticale.

Farmaci concomitanti: Tutti i farmaci concomitanti e le eventuali modifiche di terapia verranno registrati ad ogni visita.

ANALISI STATISTICA

Tutti i pazienti randomizzati che hanno assunto il farmaco saranno inclusi nell'analisi sulla sicurezza del farmaco (SF-set). Verranno inclusi nell'analisi di efficacia(EF-set) i dati dei pazienti che hanno completato lo studio senza deviazioni maggiori. Pazienti che hanno completato la visita

basale, la visita 2A e 2B (visite di valutazione 24h IOP), ma di cui mancano entrambe le visite 1A o 1B dovranno essere inclusi nell'analisi di efficacia, I pazienti randomizzati di cui mancano la visita 2A o 2B non saranno inclusi nell'analisi di efficacia, ma solo nell'analisi SF. Gli endpoint primario e secondari di efficacia saranno analizzati utilizzando i dati del EF-set. Gli endpoint di sicurezza saranno analizzati utilizzando i dati del SF-set.

I dati verranno descritti come media e come intervallo di confidenza al 95% per variabili continue e con le frequenze per le variabili categoriche.

Per le misurazione della IOP, il valore inserito nell'analisi statistica sarà la media di 2 misure in ogni orario (o la mediana di tre letture se le prime due misurazioni avevano una differenza maggiore di 2 mmHg)) Inoltre, se entrambi gli occhi soddisfano i criteri di eleggibilità, i dati dell'occhio peggiore (cioè, con i valori di pressioni intraoculare medi maggiori nelle 24 ore alla visita basale) saranno utilizzati per l'analisi.

I dati della pressione intraoculare saranno analizzati in base al trattamento e non per gruppo, in modo da mettere in comune i dati ottenuti dallo stesso trattamento a prescindere dalla sequenza di trattamento.

Un modello misto ANOVA verrà utilizzato per testare che non ci siano differenze tra pressione intraoculare media nelle 24 ore dopo 8 settimane di trattamento con Bimatoprost 0,01% e timololo 0,5% I seguenti effetti saranno inclusi nel modello: effetto della randomizzazione, l'effetto del farmaco e dell'interazione tra i farmaci, e l'effetto della sequenza di trattamento (per esplorare potenziali effetti di trascinamento). La significatività statistica è del 5%.

Sarà anche valutato il comportamento della IOP all'interno del gruppo prima e dopo il trattamento

In caso di dati mancanti di una delle due fasi di trattamento sarà effettuata un'analisi ristretta di un modello misto, valutando gli effetti del periodo e del trattamento e una matrice di covarianza simmetrica verrà eseguita sulla EF-set per l'endpoint primario

Le variabili categoriche, come le proporzioni e le variabili di sicurezza saranno analizzate utilizzando il test Chi-square di Pearson o test di Fisher a seconda dei casi.

CALCOLO DELLE DIMENSIONI DEL CAMPIONE

Un totale di 29 pazienti entreranno in questo studio crossover . La probabilità è dell'80% che lo studio rilevi una differenza di trattamento con livello di significatività di 0,05,con una differenza tra i trattamenti è di 1,5 mmHg. Questo si basa sul presupposto che la deviazione standard di risposta è di 2,0.

SEGNALAZIONE DI PROCESSI AVVERSI

I ricercatori presenteranno, entro i termini, una relazione, come per i regolamenti locali, su tutti gli eventi avversi(AEs) che si verificheranno durante il periodo di trattamento. La relazione dovrà essere presentata al comitato etico, alla struttura ospedaliera e all' AIFA.

Gli eventi avversi gravi (SAE) devono essere riportati entro le 24 ore anche al Fax: +39 06 80.956.414.

I ricercatori dovranno segnalare tutti gli eventi avversi anche sul CRF per tutta la durata dello studio.

REAZIONI AVVERSE DEL FARMACO

Una reazione avversa (ADR)del farmaco verrà registrata se si sospetta che vi sia una ragionevole possibilità che sia collegata al prodotto.

Nel caso si sospettino reazioni avverse verranno riportati alla Fondazione G.B. Bietti IRCCS, Via Livenza 3, 00198, Roma, Italia, Tel +39-6-85356727.

Se, una volta conclusosi lo studio, si presenta, un AE/SAE/ADR il paziente verrà seguito fin quando il ricercatore lo riterrà opportuno.

Le donne in gravidanza saranno seguite fino al parto o alla fine della gravidanza.

EVENTI POST-TRIAL

Si definisce AE / SAE / ADR qualsiasi evento rilevato entro 30 giorni dopo il periodo di prova o che si pensa possa essere in relazione con il trattamento.

GESTIONE DEI DATI

I dati del CRF dovranno essere inseriti in un database in cui sono garantiti sia la sicurezza sia i requisiti di coerenza. I casi con valori anomali dovranno essere ricontrollati ed eventualmente corretti

ETICITÀ , CONTROLLO DI QUALITA' E GARANZIA DI QUALITA'

La sperimentazione sarà condotta in conformità con i principi etici presenti nella nella Dichiarazione di Helsinki e nella sua modifica nel mese di ottobre 2000, Edimburgo, Scozia, negli orientamenti europei sulla buona pratica clinica (GCP) e nelle linee guida della" International Conference on Harmonisation (ICH)"

Lo sponsor garantisce il monitoraggio dei ricercatori ed il controllo connesso con la sperimentazione, gli audit, le revisioni e le ispezioni avendo accesso diretto ai dati di origine e ai documenti

Gli investigatori devono rendere accessibili a tutti i documenti originali importanti per confermare la loro coerenza con le voci dei CRF. Il monitoraggio richiederà la verifica della presenza del consenso informato, del rispetto dei criteri di inclusione / esclusione, della documentazione degli eventi avversi gravi e la registrazione dei dati che saranno utilizzati per tutte le variabili primarie e di sicurezza. Non ci saranno informazioni nei documenti di origine circa l'identità dei soggetti che dovranno essere comunicati.

CONTROLLO DI COERENZA

Il "Controllo di coerenza" sarà eseguito sui dati inseriti ; le "query " verranno rilasciate in caso di incoerenze. I dati conformi saranno validati e inseriti nel database master. Forme incoerenti saranno conservati "in sospeso" fino alla risoluzione delle contraddizioni.
