# Supplementary material for: Effects of Topical Bimatoprost 0.01% and Timolol 0.5% on Circadian IOP, Blood Pressure and Perfusion Pressure in Patients with Glaucoma or Ocular Hypertension: A Randomized, Double Masked, Placebo-Controlled Clinical Trial
Source: PLoS One. 2015 Oct 20;10(10):e0140601. doi: 10.1371/journal.pone.0140601 (PMC4615626; doi:10.1371/journal.pone.0140601)
Supplement: S2 File — (PDF) [file pone.0140601.s003.pdf]

Comparison of the effects of Bimatoprost 0.01% e Timolol 0.5% on the intraocular pressure and cardiovascular parameters

EudraCT number 2010-024272-26

Sponsor

IRCCS G.B.Bietti Eye Foundation

Via Livenza 3

00198, Roma, Italia

Principal investigator

Prof. Marco Centofanti

Glaucoma Division

IRCCS G.B.Bietti Eye Foundation

Via Livenza 3

00198, Roma, Italia

Centres

Glaucoma Division

IRCCS G.B.Bietti Eye Foundation

Via Livenza 3

00198, Roma, Italia

Investigator: Dr. Francesco Oddone

INTRODUCTION

Bimatoprost 0.03% is a prostamide analogue effective in reducing the intraocular pressure (IOP) and it is able to reduce the IOP of about 33% (31-35%) during the day and of about 28% (29-34%) during the night.

It has been recently introduced in the market a new formulation of bimatoprost at the concentration of 0.01% and no data are currently available about its efficacy and safety over the 24 hours.

The new formulation of Bimatoprost 0.01% contains a lower concentration of active principle and this has been related to a better local tolerability profile, and specifically to a lower incidence of conjunctival hyperemia, with mantained ocular hypotensive efficacy.

The purpose of this study is to investigate the ocular hypotensive efficacy, the tolerability and the effects on blood pressure and heart rate of the new formulation of bimatoprost 0.01% and compare them with those of timolol 0.5%, one of the most widely used first line drugs to treat glaucoma.

STUDY DESIGN

Prospective, randomized, placebo controlled, double masked, cross-over clinical trial

STUDY POPULATION

Ocular hypertensive and open angle glaucoma patients will be recruited for this study. Patients will be either naive to ocular hypotensive medications or in case of ongoing treatments they will undergo adequate washout. Patients naive to ocular hypotensive medications will have to show untreated IOP that, according to the investigators, could be controlled with a topical monotherapy.

MATERIALS AND METHODS

Both ocular hypertensive subjects and glaucoma subjects can be enrolled in this study.

Inclusion criteria

- Diagnosis of primary open angle glaucoma or ocular hypertension based on the 4th edition of the European Glaucoma Society Guidelines criteria.
- IOP <27 mmHg for untreated newly diagnosed patients
- IOP<22 mmHg for patients on treatment with any hypotensive medication in monotherapy and <27 mmHg after adequate washout.
- Washout IOP have to be >21 mmHg for ocular hypertension subjects while no lower limit of untreated IOP is set for glaucoma patients.

Exclusion criteria

- Age < 18 years
- Any past or active ocular disease other than glaucoma,
- Closed/barely open anterior chamber angle or history of acute angle closure

- Any cause of secondary elevation of IOP
- Argon or selective laser trabeculoplasty within the last six months
- Any previous ocular surgery, ocular inflammation/infection occurred within three months prior to the pre-trial visit
- Uncontrolled systemic diseases that might require initiating or altering concomitant use of systemic medications that can interfere with the study drugs or with IOP (e.g. beta-adrenergic antagonists, alpha-adrenergic agonists, calcium channel blockers, ACE inhibitors and angiotensin II receptor antagonists)
- Hypersensitivity to benzalkonium chloride or to any other component of the trial drug solutions
- Contraindications to the study drugs including reactive airway disease, chronic obstructive pulmonary disease, sinus bradycardia, second or third degree atrioventricular block, overt cardiac failure, or cardiogenic shock, severe allergic rhinitis and bronchial hyperreactivity.
- Pregnancy, nursing or childbearing potential without using adequate contraception.
- Participation in a clinical trial in the 3 months before the screening visit for the present study.

STUDY PLAN AND RANDOMIZATION

Enrolled patients after having performed the untreated baseline 24-hour curve of IOP and blood pressure will be randomly assigned according to a 1:1 computer-generated randomization code list to one of the 2 treatment groups.

Possible treatments will be either bimatoprost 0.01% at night plus placebo in the morning or timolol 0.5% twice (morning and evening).

After 8 weeks of treatment patients will undergo a on-treatment 24-hour IOP and blood pressure curve and will be then crossed-over to the opposite treatment so that patients trated with bimatoprost 0.01% at night plus placebo in the morning will start treatment with timolol 0.%t twice daily and vice versa. After 8 additional weeks (16 weeks from baseline) a third 24-hour assessment of IOP and blood pressure will be performed.

WASHOUT

Washout time varied according to the ongoing treatment and was 6 weeks for prostaglandin/prostamide analogues, 4 weeks for beta-blockers and alpha-agonists, 2 weeks for carbonic anhydrase inhibitors and miotics.

STUDY ENDPOINTS

Primary endpoint: comparison of the mean 24-hour IOP after 8 weeks of treatment with bimatoprost 0.01% once at night plus placebo once in the morning with that after 8 weeks of treatment with timolol 0.5% twice daily.

Secondary endpoints: the comparison of the mean IOP, BP and HR at each of the 6 timepoints of the 24h curve, the comparison of the mean day (average of the 8:00, 12:00, 16:00 and 20:00 o'clock timepoints) and night (average of the 00:00 and 04:00 o'clock timepoints) IOP, BP and HR, and the comparison of the incidence of adverse events. Additionally the mean 24h systolic and diastolic ocular perfusion pressures (OPP) will be calculated and compared between groups. The ocular perfusion pressure will be calculated as the difference between either the mean 24h systolic or the mean 24h diastolic blood pressure and the mean 24h IOP.

SCHEDULE OF THE ACTIVITIES

At baseline enrolled patients were admitted and performed a 24h IOP, BP and HR curve and were then randomized, according to a 1:1 computer-generated randomization code list, to be treated with either bimatoprost 0.01% administered once at night (08.00pm) + placebo administered once in the morning (at 8.00am) or timolol 0.5% administered twice daily (8.00am and 8.00pm). After 8 weeks of treatment a second 24h IOP, BP, and HR curve was performed and then patients were crossed-over to the opposite treatment (patients on bimatoprost 0.01% + placebo switched to timolol 0.5% twice daily and vice versa) and were followed-up for 8 additional weeks. At the end of the follow-up (16 weeks from baseline and 8 weeks after the cross-over) a third 24h IOP, BP and HR assessment was performed. There was no wash-out between the two treatment phases. The treatment duration in each phase of the study was 8 weeks and long enough to avoid any carry-over effects between drugs. In fact the washout time for topical beta-blockers is known to be 4 weeks and for prostaglandin analogues 6 weeks.

At baseline, week 8 and week 16, patients were admitted and IOP was measured by applanation tonometry at 6 time points over the 24h (in the sitting position by Goldmann applanation tonometry at 8:00, 12:00, 16:00 and 20:00 o'clock, and in the supine position by Perkins

applanation tonometry at 00:00 and 04:00 o'clock). Two additional safety visits were performed at week 4 and 12 and during these visits IOP was measured in the sitting position by Goldmann applanation tonometry at 12.00 o'clock.

The mean of two readings or the median of three readings in case of differences >2 mmHg was reported for the analysis and the same calibrated Goldmann applanation tonometer was used by the same trained investigator in each center throughout the study.

At baseline visit patients' ophthalmic and systemic history was recorded and gonioscopy, pachymetry, and standard automated acromatic perimetry, using the Humphrey 24-2 SITA Standard program, were performed. In addition to the IOP measurements a complete ophthalmological evaluation was performed at baseline and at each follow-up visits including best corrected visual acuity measurement, slit lamp examination, ophthalmoscopy and adverse events recording. At baseline and at week 8 and 16 systolic and diastolic BP and HR have been measured at each of the 6 time points of the 24h curve before the IOP measurements by the same trained investigator. Heart rate was measured manually at the radial artery by counting number of beats in 1 minute and blood pressure was measured manually by sphygmomanometry and it was reported as the mean value of three repeated measurement taken at each timepoint.

Additionally patients were asked to give specific answers to the following 3 questions at each follow-up visits: 1) did you notice any redness in your eyes since last visit? 2) did you experience any dry eye or foreign body sensation since last visit? 3) did you experience any eye burning sensation since last visit?

STUDY EYE

If only one eye was eligible as trial eye, that eye was included. If both eyes were eligible only the eye with highest baseline mean 24h IOP was included in the statistical analysis.

SUBJECTS EXCLUSION

Enrolled subjects will be excluded from the study in case of pregnancy, medical reasons at the judgement of the investigator (e.g. changes in concomitant ongoing systemic medications that could interfere with IOP), if the patients decide to terminate the study, in case of no response to the topical hypotensive treatment defined as a IOP>24 mmHg at any visit after randomization.

STUDY MEDICATIONS

Two masked sets of study medications have been prepared, one set containing bimatoprost 0.01% for the evening administration and placebo for the morning administration and one set containing two bottles of timolol 0.5%, one for the morning and one for the evening administration. Masking, randomization list generation, labelling, blinding, packaging, and shipping of trial medications was handled by Pierrel Research IMP s.r.l..

At baseline, after randomization, 1 masked box containing 3 sets of study medications corresponding to treatment-phase 1 was dispensed to each randomized patient. At the week 8 visit the medications were collected from the patients and a second box containing 3 sets of study medications corresponding of treatment-phase 2 was dispensed to each patient.

During the admissions at baseline and at the week 8 and 16 visits the masked study drugs were administered according to the schedule by the study investigators.

INTRAOCULAR PRESSURE MEASUREMENT

The IOP will be measured at 8:00, 12:00, 16:00, 20:00, 0:00, 4:00, 8:00 and only at 12.00 during the safety visits 1° and 1B. A maximum deviation of +/-15 minutes from the time schedule is allowed. IOP will be measured by Goldmann applanztion tonometry and will be reported as the average of two readings or as the median of three readings if the first two readings differ more that 2 mmHg. Only 1 calibrated tonometer will be used throughout the study.

COMPLIANCE WITH THE STUDY DRUGS

A clear explanation to each patient about when and how to use the study medications will be performed by the study investigators before the study beginning. Patients will be asked at each visit about the compliance had with the treatment schedule since the previous visit.

CONCOMITANT MEDICATIONS

During the study no other concomitant ocular topical medications are allowed. Changes of concomitant medications that, at the judgement of the investigator, can interfere with the IOP (e.g. calcium channels blockers, ACE-inhibitors ecc.). Any concomitant medications have to be recorded in the study CRF.

SAFETY

Best corrected visual acuity (BCVA) will be checked at each study visit. Any clinically relevant reduction of BCVA will be reported as an adverse event.
Slit lamp biomicroscopy will be performed at each study visit to assess any clinically relevant change in the ocular adnexes or in the eye. Specifically will be evaluated the periocular skin pigmentation, the eyelashes status, the presence of objective and self reported conjunctival hyperemia, the presence of pigment deposits on the corneal endothelium or the presence of cells and flare in the anterior chamber that will be graded as light=1(3-5 cells) moderate=2 (6-20 cells) and severe= 3 (>20 cells).
Indirect ophthalmoscopy will be performed at baseline and at the last study visist to evaluate the retina and the optic nerve status.

STATISTICAL ANALYSIS

If only one eye was eligible as trial eye, that eye was included. If both eyes were eligible only the eye with highest baseline mean 24h IOP was included in the statistical analysis.
Only patients completing the baseline, 8 weeks and 16 weeks visits (24h IOP assessment visits) were included in the efficacy analysis. All randomized patients taking any amount of the study medication were included in the safety analysis.
Data have been described as mean and 95% confidence interval (CI) for continuous variables and frequencies for categorical variables. IOP data have been analysed by treatment and not by group, thus pooling data obtained from the same treatment irrespectively of the treatment sequence. A multivariate ANOVA model for repeated measures was used to test the null hypotesis that the mean 24h IOP between treatments is not different. The treatment sequence (either first bimatoprost 0.01% + placebo or first timolol 0.5% twice daily) was included in the model as fixed effect to test the interaction between drug and treatment sequence and to explore the presence of potential carry-over effects. Post-hoc between treatments comparisons have been performed by paired t-test or Wilcoxon sign rank test after normality check performed by Shapiro-Wilk test. Categorical variables such as proportions and safety variables have been analyzed using the Pearson Chi-squared test or Fisher exact test as appropriate. Statistical significance was set at the 5% level.

SAMPLE SIZE CALCULATION

It has been estimated that the probability is 80% that a sample size of at least 29 subjects in this cross-over study will allow to detect a between treatments difference at a two-sided 5% significance level if the true difference is 1.5 mmHg or greater. This is based on the assumption that the within-patient standard deviation of the response variable is 2 mmHg.

REPORTING OF ADVERSE EVENTS

All adverse events will be reported in the study CRFs and the investigators will present within the proper time-frame a report about all adverse events that are occured during the study. This report will be presented at the ethical committee and at the AIFA.

Serious adverse events will be promptly reported within 24-hours from the event itself by fax at the number +39 06 80.956.414.

DRUG RELATED ADVERSE EVENTS

Any adverse reaction will be recorded as suspected to be related to the study drugs (ADR) if there is a reasonable possibility that the realtionship does exist. Any ADR will be comunicated to the IRCCS Fondazione Bietti at the number +39-6-85356727.

POST TRIAL EVENTS

Post trial events are defined as events that occur with 30 days after the study end. Patients with potential ADR that occurs after the study end will be followed-up until the events resolves or at the judgement of the investigators. Any pregnant women will be followed-up until delivery.

DATA MANAGEMENT

CRF data will be input in a master database after validation whose integrity and consistency will be warranted.

ETHICAL ISSUES

The study will be conducted according with the ethical principles of the declaration of Helsinky and its update of october 2000, Edimburg, Scotland, UK, with the Good Clinical Practice (GCP) guidelines and with the guidelines of the ” International Conference on Harmonisation (ICH)”.

The sponsor IRCCS Fondazione G.B.Bietti warrant the monitoring of the research activity and the control realted with this study, the audits, revisions and inspections having direct access to the source documents. The researchers have to make accessible all original study documents to confirm the consistency with the CRF data and the signature of the informed consent by th eenrolled subjects.

CONSISTENCY CONTROL

Consistency control will be performed on th entered data. The query will be released in case of inconsistencies. Consistent data will be validated and input in the master database.
